# Supplementary material for: Motion-corrected eye tracking improves gaze accuracy during visual fMRI experiments
Source: Nat Commun. 2025 Dec 18;17:1022. doi: 10.1038/s41467-025-67767-5 (PMC12848023; doi:10.1038/s41467-025-67767-5)
Supplement: Supplementary file 1 — Supplementary Information [file 41467_2025_67767_MOESM1_ESM.pdf]

Supplementary Information

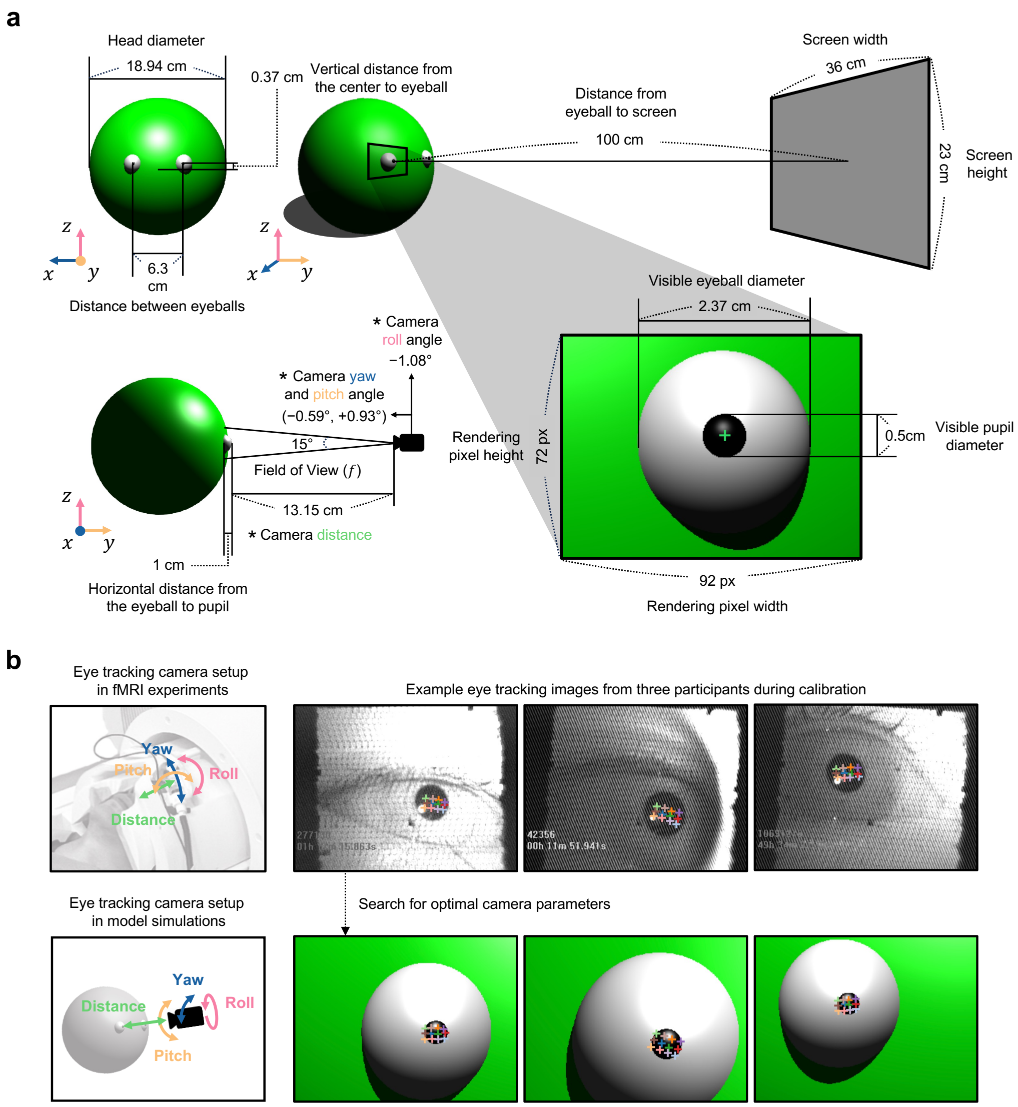

Supplementary Figure 1. Anthropometric parameters used in the model simulation. a) The head model was designed as an isotropic sphere to simplify head motion dynamics, with the diameter defined by averaging three key human head dimensions: head breadth (ear-to-ear distance), horizontal depth (back of head to nose), and vertical length (chin to top of head), based on measurements from prior studies[1-3]. The model's eyeballs are physically attached to the head sphere, ensuring that head movements directly influence the 3D spatial position of the eyeballs. Each eyeball can independently rotate to simulate gaze shifts, while the pupil, aligned with the gaze direction, is positioned along the line extending from the eyeball center to the target location. For visualization purposes, the example model eye was rendered at a high-resolution of 512×384 pixels, whereas the actual simulation was conducted at 92×72 pixels for computational efficiency. **b)** To replicate realistic eye tracking conditions, virtual camera parameters, including yaw, pitch, roll, and distance from the eyeball, were customized to match participant-specific eye tracking data. A total of 36,000 parameters configurations were tested by simulating gaze toward 12 calibration points on the screen. The optimal camera parameters were selected for each individual eye tracking data by minimizing the discrepancy between simulated and actual participant pupil coordinates, ensuring the simulated eye tracking data closely aligned with participant data. The bottom panels show the optimized camera parameters and corresponding simulated pupil locations for 12 calibration points, illustrated for three example participants shown in the top panels. Averaged values of these camera parameters across participants are indicated with an asterisk (*) in panel **a**.

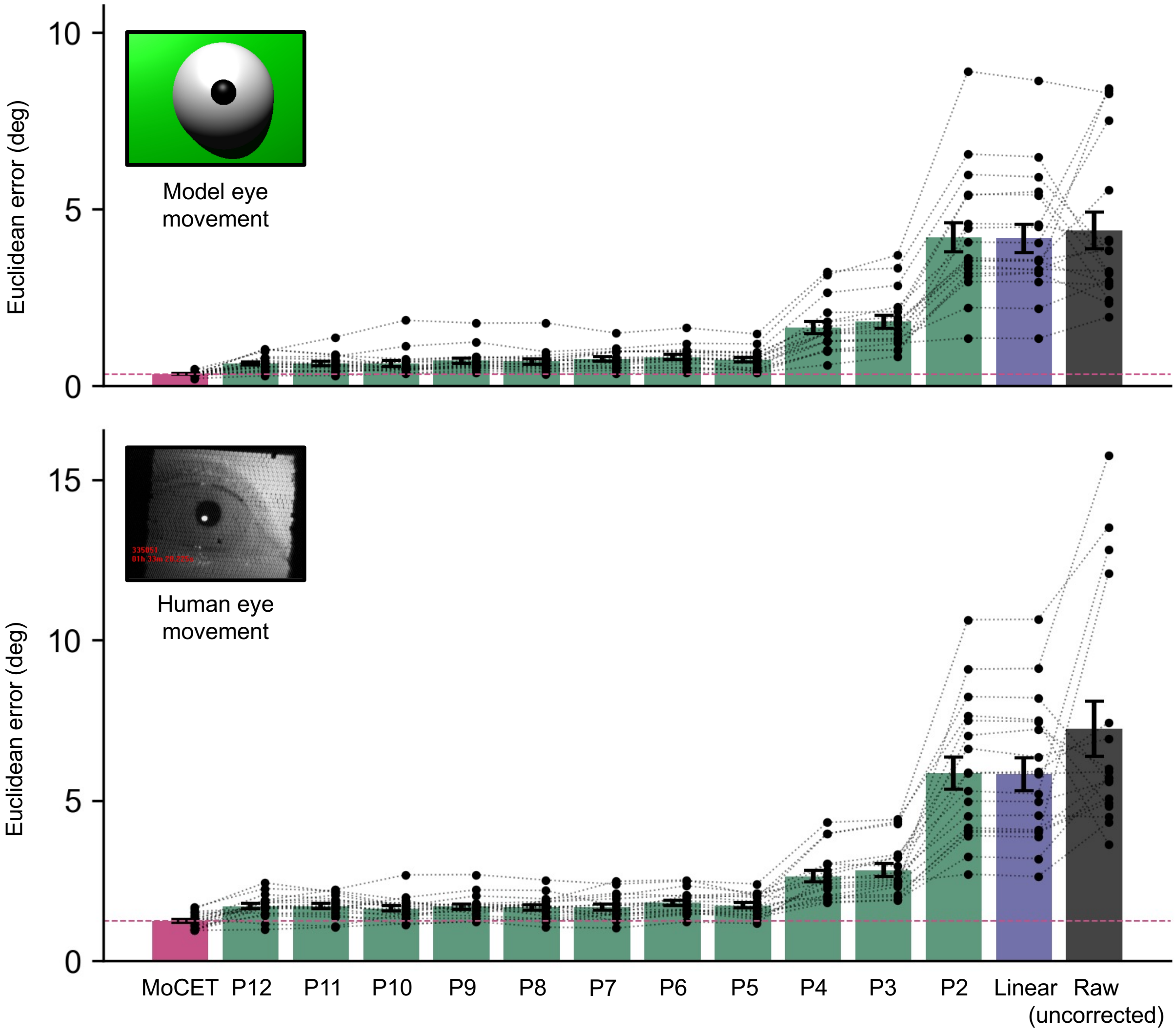

Supplementary Figure 2. Extended analysis of gaze accuracy during the validation stage for model simulation (top) and human eye tracking data (bottom) from Fig. 4a. Gaze accuracy is compared across MoCET and various detrending methods, including linear detrending and polynomial detrending up to the 12th order. Polynomial methods show diminishing returns in performance improvement beyond the 5th order, while MoCET consistently achieves the highest accuracy across both datasets. Horizontal dashed lines indicate MoCET's performance as a benchmark Euclidean errors were compared across methods using paired t-tests with two-sided p-values. Error bars indicate ±1 s.e.m. across participants ($N = 18$). Source data are provided as a Source Data file.

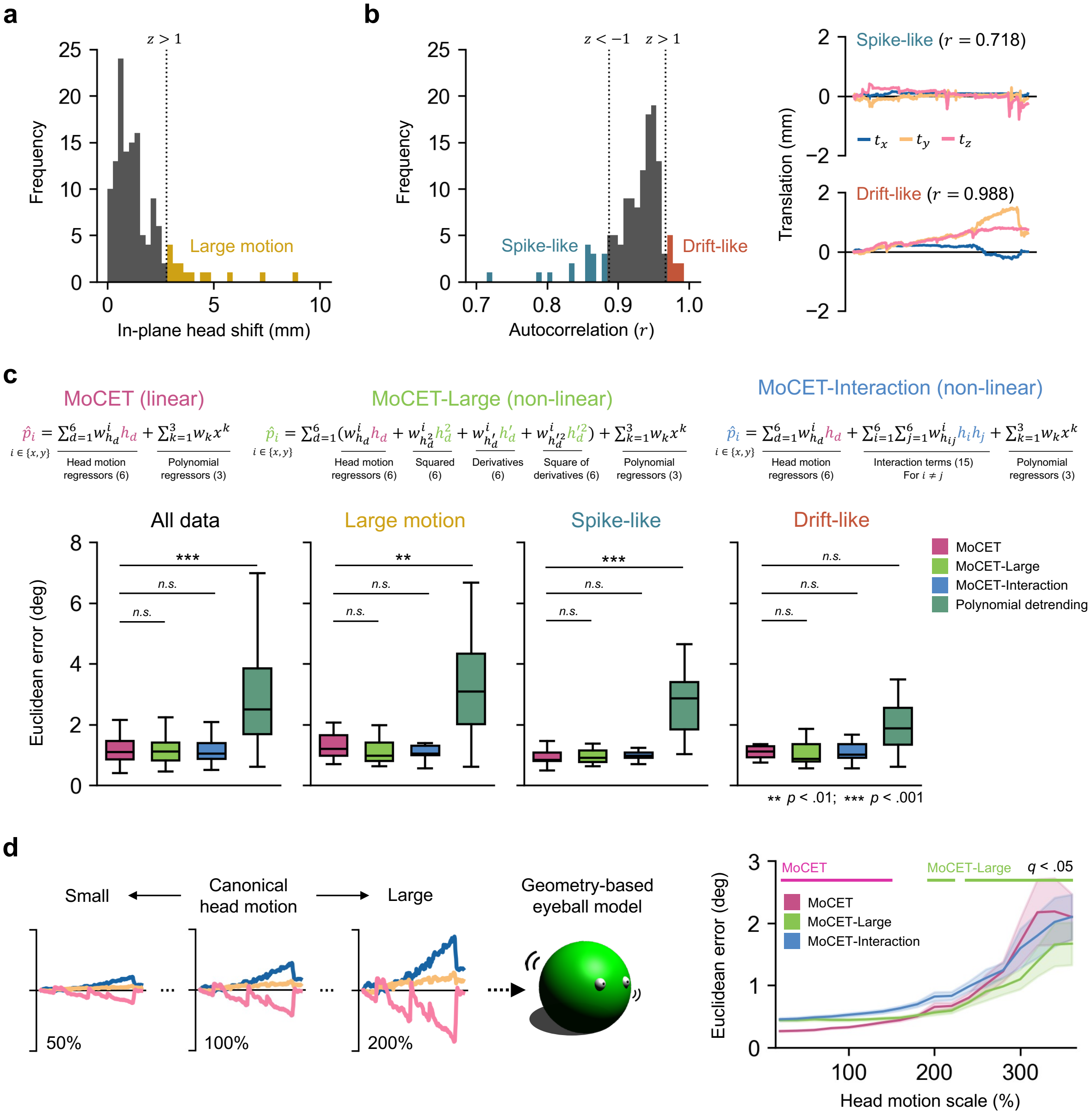

Supplementary Figure 3. Characterizing head motion types and evaluating nonlinear variants of MoCET. a) Histogram of in-plane head motion magnitude across runs. Runs with z-scored magnitude > +1 were classified as *large motion* ($N = 13$). **b)** Histogram of motion autocorrelation. Runs with z-scored autocorrelation > +1 were classified as *drift-like* ($N = 12$), and those with z-scored autocorrelation < −1 as *spike-like* ($N = 16$). Example translational motion traces from each type are shown. **c)** Gaze correction accuracy across methods: linear MoCET, nonlinear variants (MoCET-Large and MoCET-Interaction), and polynomial detrending. Across all motion types (all runs, large motion, spike-like, drift-like), nonlinear variants did not significantly outperform linear MoCET. Polynomial detrending was comparable to MoCET only in drift-like runs and was otherwise less effective. **d)** Simulated head motion amplitude scaling analysis. As motion magnitude increased from 20% to 140% of the original, linear MoCET consistently outperformed nonlinear variants. Under extreme motion (>200%), MoCET-Large outperformed standard MoCET. Shaded areas indicate ±1 s.e.m. computed from all eye tracking data across all participants. Source data are provided as a Source Data file.

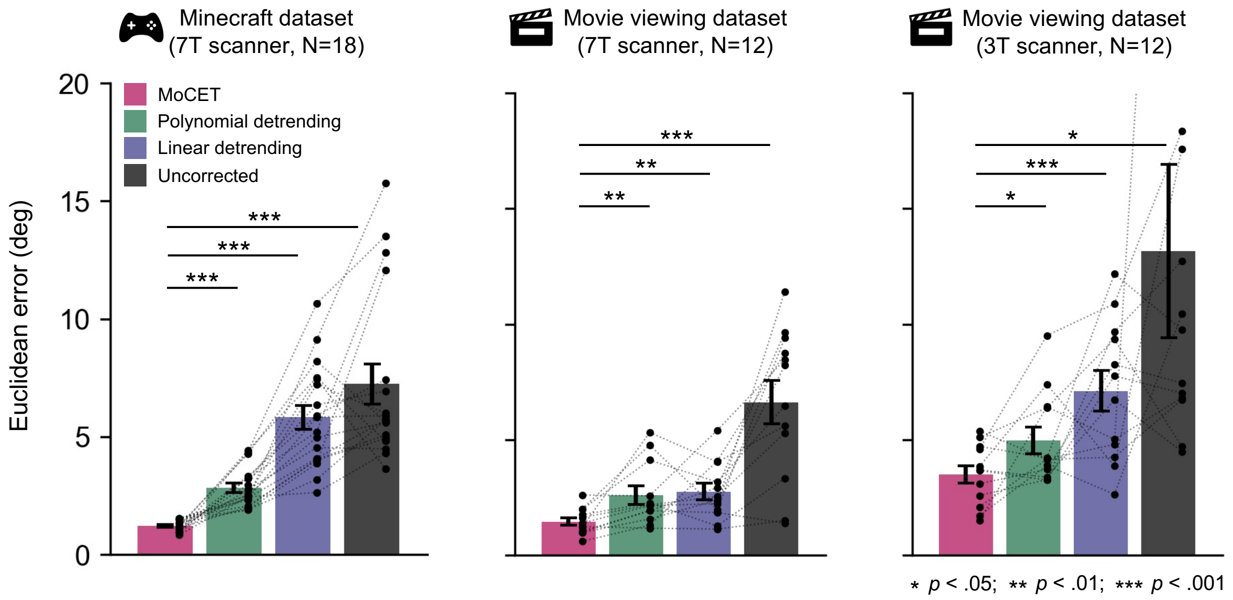

Supplementary Figure 4. Comparison of eye tracking correction performance across datasets. Gaze accuracy is compared among MoCET, polynomial detrending, linear detrending, and uncorrected data across three independent datasets: video game play (7T), movie watching (7T), and movie watching (3T). MoCET consistently outperforms uncorrected data and conventional detrending methods across tasks and scanners. Euclidean errors were compared across methods using paired t-tests with two-sided *p*-values. Error bars indicate ±1 s.e.m. across participants (video game play, $N = 18$; movie watching, $N = 12$). Source data are provided as a Source Data file.

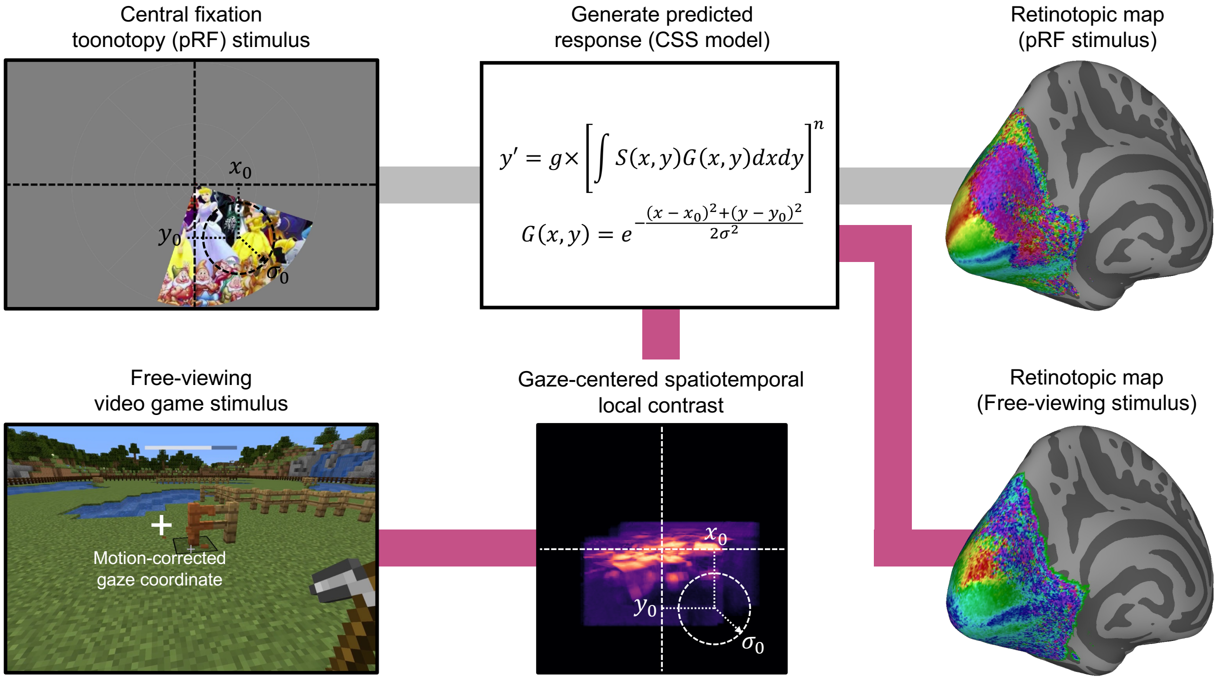

Supplementary Figure 5. Estimation of retinotopic mapping from pRF experiments and free-viewing video game stimuli. Retinotopic mappings in early visual areas were derived using structured pRF stimuli with central fixation (top) and free-viewing video game stimuli (bottom). For pRF experiments, structured stimuli[4] such as wedges, rings, and bars were presented while participants maintained central fixation. Neural responses were modeled using the Compressive Spatial Summation (CSS) model[5] to estimate retinotopic map across the visual cortex. For free-viewing experiments, detrended eye tracking data (e.g., MoCET or polynomial detrending) was used to transform visual stimuli into gaze-centered spatiotemporal local contrast maps[6], which served as input to the pRF model. The video game screenshot is used under the Minecraft Usage Guidelines (©2025 Mojang AB; Minecraft® is a trademark of Microsoft Corporation).

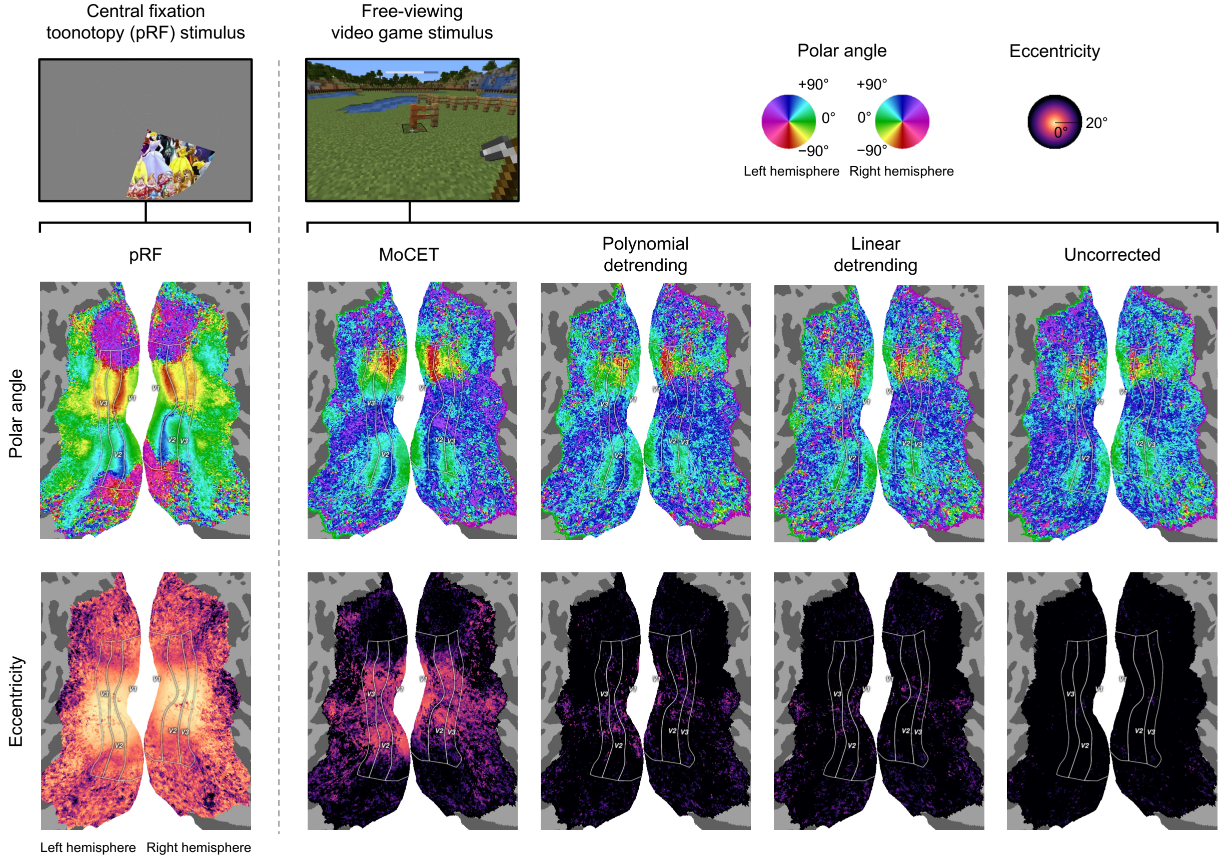

Supplementary Figure 6. Group-averaged retinotopic maps estimated from pRF experiments and free-viewing visual stimuli. Flattened cortical maps display polar angle retinotopy in early visual areas (V1, V2, V3) for both hemispheres. Visual field maps are derived using structured pRF experiments with central fixation (pRF) and free-viewing video game stimuli corrected with MoCET, polynomial detrending, linear detrending, or uncorrected data. Regions of interest (ROIs) for V1, V2, and V3 were defined based on the Human Connectome Project (HCP) retinotopy dataset[7]. The video game screenshot is used under the Minecraft Usage Guidelines (©2025 Mojang AB; Minecraft® is a trademark of Microsoft Corporation).

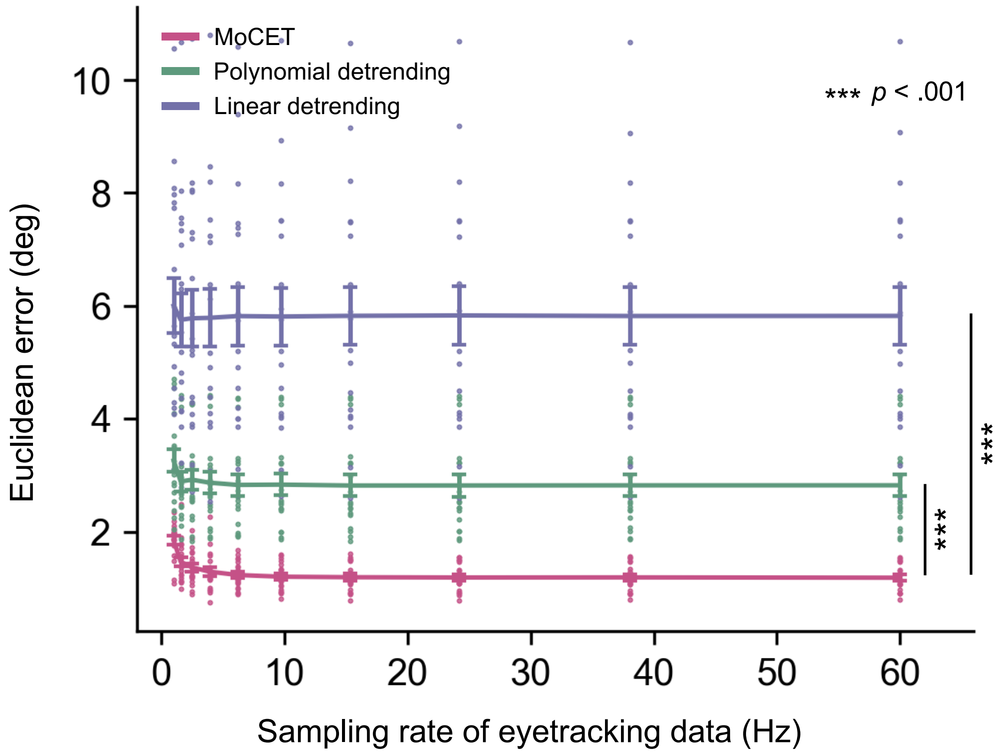

Supplementary Figure 7. Impact of eye tracking sampling rate on correction accuracy. Eye tracking data were downsampled from 60 Hz to 1 Hz to assess how temporal resolution affects correction accuracy. MoCET maintained high accuracy down to 10 Hz and continued to outperform other detrending methods even at lower sampling rates, demonstrating robustness to temporal resolution mismatch. Euclidean errors were compared across methods using paired t-tests with two-sided *p*-values. Error bars indicate ±1 s.e.m. across participants. Source data are provided as a Source Data file.

Supplementary References

1. Rashid, A. B. & Showva, N.-N. Design and fabrication of a biodegradable face shield by using cleaner technologies for the protection of direct splash and airborne pathogens during the COVID-19 pandemic. *Clean. Eng. Technol.* **13**, 100615 (2023).
2. Villoing, D. et al. KOREAN PEDIATRIC AND ADULT HEAD COMPUTATIONAL PHANTOMS AND APPLICATION TO PHOTON SPECIFIC ABSORBED FRACTIONS CALCULATIONS. *Radiat. Prot. Dosim.* **176**, 294–301 (2017).
3. Lee, W. et al. A 3D anthropometric sizing analysis system based on North American CAESAR 3D scan data for design of head wearable products. *Comput. Ind. Eng.* **117**, 121–130 (2018).
4. Finzi, D. et al. Differential spatial computations in ventral and lateral face-selective regions are scaffolded by structural connections. Nat. Commun. 12, 2278 (2021).
5. Kay, K. N., Winawer, J., Mezer, A. & Wandell, B. A. Compressive spatial summation in human visual cortex. J. Neurophysiol. **110**, 481–494 (2013).
6. Allen, E. J. et al. A massive 7T fMRI dataset to bridge cognitive neuroscience and artificial intelligence. *Nat Neurosci* **25**, 116–126 (2022).
7. Benson, N. C. et al. The Human Connectome Project 7 Tesla retinotopy dataset: Description and population receptive field analysis. J. Vis. 18, 23 (2018).
