# Supplementary material for: Motion-corrected eye tracking improves gaze accuracy during visual fMRI experiments
Source: Nat Commun. 2025 Dec 18;17:1022. doi: 10.1038/s41467-025-67767-5 (PMC12848023; doi:10.1038/s41467-025-67767-5)
Supplement: Supplementary file 2 — Reporting Summary [file 41467_2025_67767_MOESM2_ESM.pdf]

nature portfolio

Corresponding author(s): Won Mok Shim

Last updated by author(s): Nov 28, 2025

Reporting Summary

Nature Portfolio wishes to improve the reproducibility of the work that we publish. This form provides structure for consistency and transparency in reporting. For further information on Nature Portfolio policies, see our Editorial Policies and the Editorial Policy Checklist.

Statistics

For all statistical analyses, confirm that the following items are present in the figure legend, table legend, main text, or Methods section.

n/a	Confirmed	
☐	☒	The exact sample size (*n*) for each experimental group/condition, given as a discrete number and unit of measurement
☐	☒	A statement on whether measurements were taken from distinct samples or whether the same sample was measured repeatedly
☐	☒	The statistical test(s) used AND whether they are one- or two-sided<br>*Only common tests should be described solely by name; describe more complex techniques in the Methods section.*
☐	☒	A description of all covariates tested
☐	☒	A description of any assumptions or corrections, such as tests of normality and adjustment for multiple comparisons
☐	☒	A full description of the statistical parameters including central tendency (e.g. means) or other basic estimates (e.g. regression coefficient) AND variation (e.g. standard deviation) or associated estimates of uncertainty (e.g. confidence intervals)
☐	☒	For null hypothesis testing, the test statistic (e.g. F, t, r) with confidence intervals, effect sizes, degrees of freedom and P value noted<br>*Give P values as exact values whenever suitable.*
☒	☐	For Bayesian analysis, information on the choice of priors and Markov chain Monte Carlo settings
☒	☐	For hierarchical and complex designs, identification of the appropriate level for tests and full reporting of outcomes
☐	☒	Estimates of effect sizes (e.g. Cohen's *d*, Pearson's *r*), indicating how they were calculated

Our web collection on statistics for biologists contains articles on many of the points above.

Software and code

Policy information about availability of computer code

Data collection	Data collection was conducted using Minecraft (version 1.14.4), developed by Mojang Studios, to present the virtual environment and interactive tasks. Eye-tracking data were recorded using ViewPoint EyeTracker software (version 2.9.5.111) by Arrington Research.
Data analysis	Neuroimaging data were preprocessed using fMRIprep (version 21.0.2) for head motion correction and anatomical registration. Eye-tracking data were preprocessed using EyeRec and custom-written Python scripts. Subsequent analyses for both eye-tracking and neuroimaging data were performed using Python (version 3.11.4), with the following libraries: numpy, scipy, and scikit-learn.

For manuscripts utilizing custom algorithms or software that are central to the research but not yet described in published literature, software must be made available to editors and reviewers. We strongly encourage code deposition in a community repository (e.g. GitHub). See the Nature Portfolio guidelines for submitting code & software for further information.

Data

Policy information about availability of data

All manuscripts must include a data availability statement. This statement should provide the following information, where applicable:

- Accession codes, unique identifiers, or web links for publicly available datasets
- A description of any restrictions on data availability
- For clinical datasets or third party data, please ensure that the statement adheres to our policy

We have publicly shared the dataset (https://zenodo.org/records/17089244) and code (https://github.com/jwparks/mocet), ensuring reproducibility for peer review and publication.

Research involving human participants, their data, or biological material

Policy information about studies with human participants or human data. See also policy information about sex, gender (identity/presentation), and sexual orientation and race, ethnicity and racism.

Reporting on sex and gender	A total of 19 participants (8 females and 11 males) were recruited for the study, of whom 18 participants met the data inclusion criteria. No sex/gender-related analyses were performed.
Reporting on race, ethnicity, or other socially relevant groupings	The participants in this study were all Asian, specifically Korean. No race/ethnicity-related analyses were performed.
Population characteristics	The mean age of the participants was 24.02 years (s.d. = 2.54 years).
Recruitment	Participants in this study were recruited from the the Sungkyunkwan University online community.
Ethics oversight	The study was approved by the Sungkyunkwan University Institutional Review Board.

Note that full information on the approval of the study protocol must also be provided in the manuscript.

Field-specific reporting

Please select the one below that is the best fit for your research. If you are not sure, read the appropriate sections before making your selection.

☐ Life sciences ☒ Behavioural & social sciences ☐ Ecological, evolutionary & environmental sciences

For a reference copy of the document with all sections, see nature.com/documents/nr-reporting-summary-flat.pdf

Behavioural & social sciences study design

All studies must disclose on these points even when the disclosure is negative.

Study description	This study introduces Motion-corrected eye tracking (MoCET), a new method that enhances the spatial accuracy of camera-based eye tracking during fMRI experiments by correcting drift induced from head motion. By using motion parameters obtained from fMRI preprocessing pipelines, MoCET enables retrospective correction without the need for additional hardware.
Research sample	Nineteen participants (8 females; mean age = 24.02 ± 2.54 years) were recruited from the Sungkyunkwan University community. All participants had normal or corrected-to-normal vision, and gave written informed consent in accordance with institutional IRB approval. Data from twenty participants were included in the final analysis after quality control.
Sampling strategy	Convenience sampling was used. The sample size was determined based on prior fMRI studies involving naturalistic tasks, as well as logistical considerations for data collection and analysis.
Data collection	Eye tracking and fMRI data were collected simultaneously while participants performed free-viewing, Minecraft-based cognitive task in a 7T MRI scanner. Eye movements were recorded using an Avotec endoscopic camera system at 60 Hz. Additional fMRI sessions using conventional population receptive field (pRF) mapping were collected for all 18 participants. Data collection and preprocessing followed standard procedures detailed in the Methods section.
Timing	The data were collected from January 5, 2023, to January 10, 2025.
Data exclusions	One participant was excluded from analysis due to not meeting validation criteria for eye tracking accuracy in any runs. Only runs with gaze errors <1.0° visual angle during both calibration and validation were retained, leading to inclusion of 18 participants with an average of 7.39 ± 3.03 verifiable runs each.
Non-participation	No participants withdrew from the study after enrollment and consent.
Randomization	Randomization was not applicable, as all participants underwent the same task protocol.

Reporting for specific materials, systems and methods

We require information from authors about some types of materials, experimental systems and methods used in many studies. Here, indicate whether each material, system or method listed is relevant to your study. If you are not sure if a list item applies to your research, read the appropriate section before selecting a response.

Materials & experimental systems

n/a	Involved in the study
x	Antibodies
x	Eukaryotic cell lines
x	Palaeontology and archaeology
x	Animals and other organisms
x	Clinical data
x	Dual use research of concern
x	Plants

Methods

n/a	Involved in the study
x	ChIP-seq
x	Flow cytometry
	x MRI-based neuroimaging

Plants

Seed stocks: n/a

Novel plant genotypes: n/a

Authentication: n/a

Magnetic resonance imaging

Experimental design

Design type: Free-viewing naturalistic paradigm using an interactive 3D Minecraft-based task

Design specifications: Participants completed either one or two fMRI sessions. Each session consisted of six runs of a Minecraft-based interactive cognitive task. At the beginning of each run, participants performed a calibration task involving fixation on 24 targets presented sequentially; at the end of each run, a validation task with 12 fixation targets was presented. Each run lasted 13 minutes and 36 seconds. Eye tracking data were recorded throughout each run at 60 Hz, and fMRI volumes were acquired with a TR of 1.6 second.

Behavioral performance measures: Eye tracking data were continuously recorded during the task. In addition, in-game behavior was logged through event files capturing the timing and location of all player actions (e.g., fence construction or removal). These logs were used to identify the visual target associated with each action. A behavioral performance metric ("hit rate") was computed by measuring the proportion of time participants' gaze fell within 2 degrees of visual angle from the target location during the 10 seconds preceding each action. This hit ratio was used to assess the relevance of gaze behavior to the task and was compared across eye tracking correction methods using paired t-tests.

Acquisition

Imaging type(s): Functional, Structural

Field strength: 7T

Sequence & imaging parameters: Blood oxygenation level-dependent (BOLD) contrast was measured using T2*-weighted functional images obtained with a dual-polarity GRAPPA (DPG) sequence (voxel size: 1.5 mm isotropic; TR: 1600 ms; TE: 21 ms; FOV: 210 × 210 mm; 96 slices covering the whole brain; flip angle: 50°). High-resolution anatomical images were acquired using an MP2RAGE sequence (voxel size: 0.7 mm isotropic; TR: 5888 ms; TE: 2.44 ms; FOV: 224 × 224 mm; 320 slices; flip angle 1: 4°; flip angle 2: 5°).

Area of acquisition: whole-brain

Diffusion MRI: ☐ Used ☒ Not used

Preprocessing

Preprocessing software: fMRIprep (version 21.0.2; https://fmriprep.org/en/stable/) was used for correcting head motion and registering each participant's brain to the spatial normalization template.

Normalization: Spatial normalization to standard spaces was performed using ANTs' antsRegistration in a multiscale, mutual-information based, nonlinear registration scheme in the fMRIprep pipeline.

Normalization template	MNI152NLin2009cAsym template
Noise and artifact removal	None
Volume censoring	None

Statistical modeling & inference

Model type and settings	Mass univariate voxelwise modeling. A Gaussian population receptive field (pRF) model was fit separately to each voxel using gaze-centered stimulus representations derived from free-viewing video game stimuli. The spatiotemporal local contrast of gaze-centered visual input was computed and used as the model input to predict voxelwise BOLD time series.
Effect(s) tested	The model estimated the polar angle preference of each voxel in early visual cortex (V1–V3) by fitting the Gaussian receptive field to explain the voxel's response to gaze-centered spatiotemporal contrast patterns during free-viewing. Effects were compared across different drift correction methods (MoCET, polynomial, linear, and uncorrected).

Specify type of analysis: ☐ Whole brain ☒ ROI-based ☐ Both

Anatomical location(s): Early visual areas including V1, V2, and V3, in both hemispheres. ROIs and anatomical landmarks were defined using the Human Connectome Project retinotopy dataset.

Statistic type for inference (See Eklund et al. 2016)	No whole-brain analyses were performed.
Correction	No multiple comparisons correction was applied, as the primary statistical tests were performed on summary metrics (e.g., angular error) averaged within predefined regions of interest. All reported p-values reflect direct comparisons across methods at the group level.

Models & analysis

n/a	Involved in the study
☒	☐ Functional and/or effective connectivity
☒	☐ Graph analysis
☒	☐ Multivariate modeling or predictive analysis
